# Decoding nature’s grammar with DNA language models

**DOI:** 10.1073/pnas.2512889122

**Published:** 2025-07-14

**Authors:** Peter L. Morrell, Serguei V. Pakhomov

**Affiliations:** ^a^Department of Agronomy and Plant Genetics, University of Minnesota, St. Paul, MN 55108; ^b^Department of Pharmaceutical Care and Health Systems, University of Minnesota, Minneapolis, MN 55455

In this issue, Zhai et al. (1) report a “DNA language model” designed to help identify the most critical variants in DNA sequence. With only a four-letter alphabet (A, C, G, and T), most base pairs in any stretch of an individual’s DNA are identical between the maternal and paternal copies. Most sequence positions are also identical between individuals in a population or related species. But even differences at <1% positions can result in millions of DNA sequence polymorphisms across a genome, encoding the visible (and invisible) differences between individuals. Yet, most polymorphisms do not affect any trait and are evolutionarily neutral. This raises a fundamental question: Which variants affect traits and evolution?

Zhai et al. (1) leverage rapid advances in AI to implement their approach in the software they call PlantCaduceus. Their approach resembles large language models (LLMs) applied to written text. The authors compare PlantCaduceus to prior applications of machine learning to this problem (2) and earlier approaches (3, 4) built on decades of research in molecular evolution. Earlier approaches start with alignments of DNA sequences; which are often trivial in coding regions and related species. The underlying assumption is that nucleotide sites subject to selective restraint are less likely to change, particularly when function is retained over evolutionary time.

Fortunately, the assumption of conserved function is reasonable, and many variants in coding sequences are among the most likely to have positive or negative effects on organismal fitness. Alignment is challenging outside of coding sequences as the four-letter alphabet for DNA becomes “saturated” with differences, and structural variation complicates the homology inference. Widely used tools for predicting constraints in noncoding sequences, for example, GERP++ (5), work well with sufficient alignment depth. However, difficulties finding alignable sequences from other species for noncoding regions can limit these approaches’ accuracy and the genomic regions where they can be applied (6). Zhai et al. (1) and some previous efforts (2) overcome this problem by forgoing alignments and using 512-nucleotide windows with a query position at their center. They can then ask about the probability of observing any of the four DNA letters at the query position when compared across species, given the immediate context of the surrounding nucleotides. This particular approach exploits a property shared by all languages—human, computer, and DNA—predictability. One of the earliest formal observations of this property dates to the 1950s and Claude Shannon, who empirically demonstrated that native speakers of a language can reliably anticipate the next character in a sequence of printed characters that form a piece of coherent text in that language (7). The longer the sequence of characters, the easier it is to predict the next character. This and other observations in Shannon’s work on modeling language gave rise to an information-theoretic measure called “perplexity” (a derivative of “cross-entropy”), which is now widely used for training statistical language models, including the one used by Zhai et al. (1). Perplexity quantifies the extent to which a language model is “surprised” when presented with a piece of text (called a “token,” which can be a word, part of a word, or even punctuation) in the reference sequence it tries to predict. We experience this phenomenon firsthand when we tap in a text message on a smartphone and the messaging app persistently autocorrects what we type to what it “thinks” we should type—i.e., the language model driving the app does not like to be “perplexed.” It is designed to minimize its perplexity, often to the detriment of our best creative intentions. To a language model trained on DNA, encountering a polymorphism in a highly evolutionary constrained portion of the DNA would be “perplexing” in a similar way to encountering the word “mustache” instead of “kingdom” immediately following “A horse, a horse, my __ for a horse!” would be perplexing to a language model trained on the works of William Shakespeare.

**Fig. 1.**
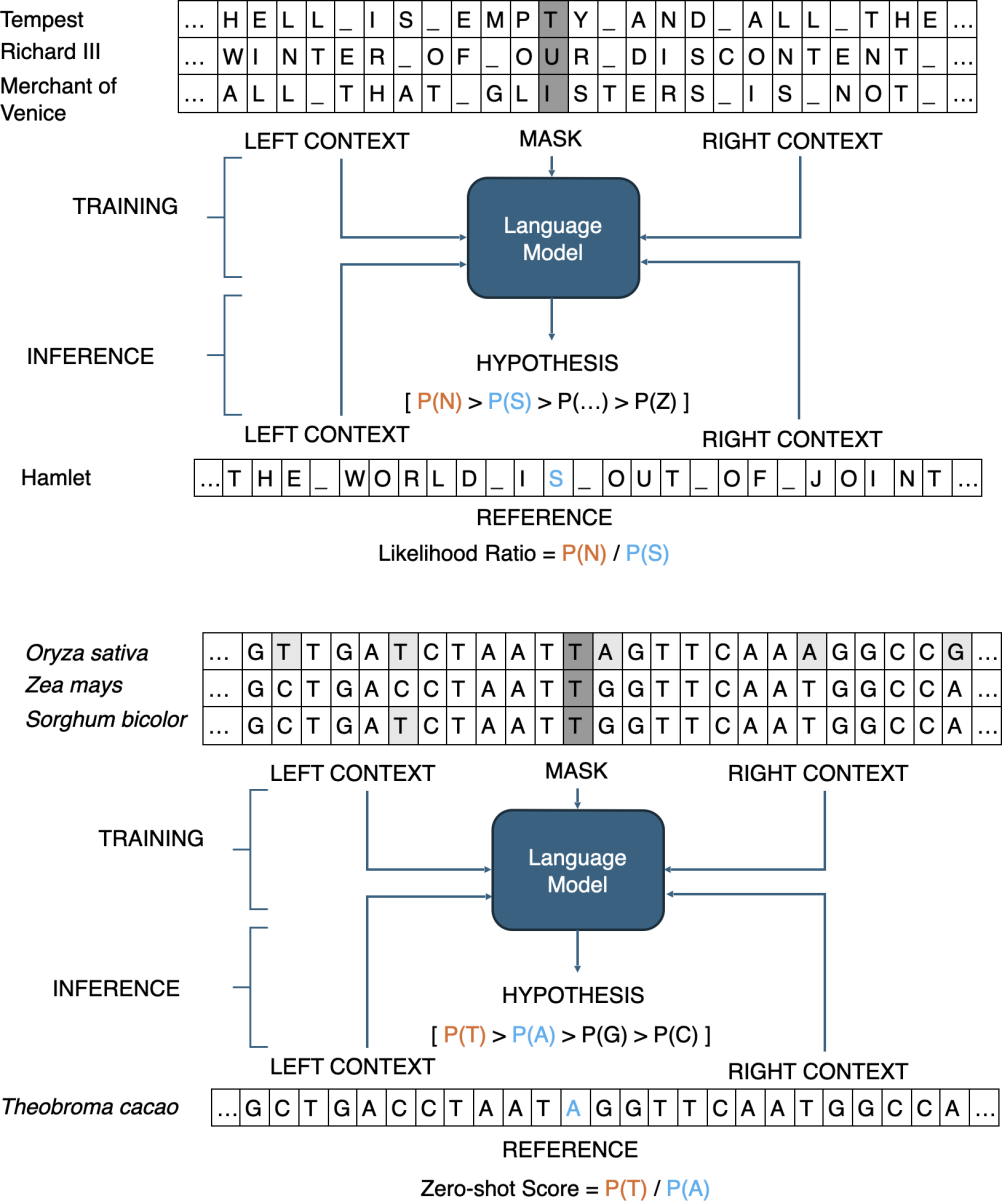
A depiction of the processes used by language models for both text and DNA sequence.

When applied to DNA sequences and freed from alignments, the foundational information-theoretic notions, such as perplexity, combined with modern, scalable neural language modeling techniques, can explore more of the genome, particularly noncoding sequences that can induce or alter gene expression. A 512-nucleotide string has 4^512^ combinations, a very large number, which, based on randomly chosen nucleotides, would be unique within a genome. However, genomes are not composed of randomly synthesized strings. Genes share features like start/stop codons, splicing signals for removing noncoding introns, and protein domains that the models can identify across genes. In addition to these functional elements, some components of the genome are duplicated nearly intact. Such duplication of genes can create copy number variation. Another major component is transposable elements—DNA that duplicates and relocates within the genome. These mobile elements constitute a large portion of most genomes, and the presence of abundant copies of the element within the genome could weight training and thus predictions, especially if the model becomes biased toward the nucleotide composition of those repetitive regions. To avoid this, the authors apply PlantCaduceus to 1,000 base pair windows around annotated genes. This is an advance over prior approaches that only attempted to annotate codons or alignable regions immediately adjacent to genes (Fig. 1). During inference, the model is provided with the context of 512 nucleotides centered on the target position and outputs probabilities for each of the four nucleotides in its vocabulary. The probability of the most likely predicted nucleotide is divided by the probability of the reference nucleotide to derive a likelihood ratio score indicating the degree to which the reference nucleotide might be deleterious.

A secondary source of constraint information is the frequency at which variants occur in populations. Harmful mutations, potentially those with the largest phenotypic effects, tend to be held to low frequencies. This count of the “site frequency spectrum” can be partitioned into classes of variants, with the most constraint at sites that disrupt gene structure, including premature stop codons, loss of start codons, and changes that alter intron and exon boundaries. Examination of the site frequency spectrum for variants queried with PlantCaduceus reveals that positions where observed variants differ most from the model's predictions occur at lower frequencies. This gold-standard comparison suggests that PlantCaduceus performs as well as, or better than, prior alignment-based approaches in predicting constraints.

DNA sequence differs from written text in other ways. It is bidirectional and, while linear, can be read in either direction. PlantCaduceus uses a recently developed novel state space model architecture, Mamba (8), designed explicitly for modeling sequences and is particularly well-suited for long sequences such as DNA. Earlier neural language model architectures applied to human text, such as the Bidirectional Encoder Representations from Transformers (BERT) (9), are bidirectional in that they can predict words based on words that occur both before and after the predicted word. BERT has also been successfully applied to genomic sequences (10). The newer Mamba approach has recently garnered attention in genomic and language modeling due to its improved computational efficiency and increased accessibility in the resource-constrained research community. The authors use cross-entropy (equivalent to perplexity) to pretrain a set of Mamba models on 16 plant genomes, employing the same random masking approach initially developed for training BERT models. This approach consists of randomly withholding approximately 15% of the letters in the training data (i.e., masking), forcing the model to predict the withheld letter (i.e., forwarding), comparing the predicted letter to the withheld letter (i.e., loss function), and subsequently adjusting the model’s parameters based on the difference between the predicted and the withheld letter (i.e., backpropagation).

In PNAS, Zhai et al. (1) report a “DNA language model” designed to help identify the most critical variants in DNA sequence.

Another important contribution of Zhai et al. is the development of an evaluation framework that leverages extensive prior work in plant genomics. The authors focus on well-characterized components of genomes, including Arabidopsis transcription initiation and termination sites, splice donor and acceptor sites, and evolutionary constraints identified through traditional cross-species alignment approaches, as well as a well-studied causal sweet corn mutation, to create benchmarks for testing their methods. The authors then used these benchmarks to compare their new approach to top-performing alignment-based and neural language modeling-based methods, thereby not only establishing a new state-of-the-art (SOTA) baseline but also paving the way for other researchers to continue advancing SOTA in this area, further assisted by publicly releasing a repository containing computer code needed to replicate the results reported in the article.

PlantCaduceus models are also transferable, so a model built without maize training data but with data from other related grasses can be applied to maize and provide useful predictions of nucleotide state (1). Researchers working on other species can bring their data, in the form of a variant call format file trimmed to regions that flank annotated genes, and produce predictions without retraining a model.

What is next for PlantCaduceus and similar approaches? First, the training dataset has always been important for older constraint-based approaches. Identifying constraints on codons works well with genomes from across the flowering plants (11). Since the primary advantage of these newer approaches is the expansion of predictions to noncoding sequences, comparison to DNA sequences from closely related species will likely be more critical than in the past. Other considerations include the size of the window chosen for comparisons. The 512-nucleotide window was chosen for convenience, but other researchers suggest that this choice may require tuning (2). Additionally, what is the optimal solution for addressing transposable elements in predictions? Accurate identification of polymorphisms in these elements is challenging because sequence reads often do not map uniquely, meaning that elements from the same portion of the genome are not being compared. It is also reasonable to ask whether selective constraints on variants in transposable elements are important; other research suggests that the selective constraint on transposable elements is roughly equal to the metabolic cost to replicate them (12).

The most exciting era for PlantCadeuceus, as well as other LLM approaches in genomics, likely lies ahead and may be ushered in by new efforts in the AI and machine learning communities aimed at improving the interpretability of these models, extending beyond the use of the perplexity measure. For example, ongoing work on using sparse autoencoders to aid in interpreting how neural LLMs represent and process information, given their billions of parameters, can be particularly relevant (13). These evolving approaches are yielding promising results, as demonstrated by enabling the identification of specific neural language model parameters that encode humanly interpretable features such as the “Golden Gate Bridge feature” which responds only to references to the Golden Gate Bridge and related concepts in the prompt, and modifying the model parameters associated with this feature results in the model starting to identify with the Golden Gate Bridge (i.e., responding as if it were the bridge!) (14). Applied to models of DNA, such as PlantCadeuceus, these approaches can potentially help interpret genetic sequences that lie outside coding regions. For example, we could potentially identify model parameters that respond to splice acceptor sites in the coding portions of the DNA and monitor these parameters when examining the noncoding DNA.

We are just beginning to unlock the potential of self-supervised LLMs and generative AI across all fields of science, including biology. It may be possible to query genomes using generative DNA language models in the same way we discuss various domains of human knowledge with models like ChatGPT. However, first we need to master the language of DNA to ask the right questions and understand the responses.
